# Poor Efficacy of Immune Checkpoint Inhibitors Plus Chemotherapy in Lung Cancer Patients with EGFR/ERBB2 Exon 20 Insertion

**DOI:** 10.3390/curroncol30110721

**Published:** 2023-11-14

**Authors:** Yue Zheng, Yang Fu, Yueyun Chen, Qing Li, Ting Liu, Zhenyu Ding

**Affiliations:** Department of Biotherapy, Cancer Center, West China Hospital, West China Medical School, Sichuan University, Chengdu 610041, China; elenazhengy8396@163.com (Y.Z.); fuyang1208@163.com (Y.F.); 13027469041@163.com (Y.C.); liqing@scu.edu.cn (Q.L.); liuting2@stu.scu.edu.cn (T.L.)

**Keywords:** EGFR, ERBB2, exon 20 insertion, immunochemotherapy, lung cancer

## Abstract

Background: EGFR and ERBB2 exon 20 insertion (Ex20ins) account for a small fraction of patients with EGFR mutations. The efficacy of immune checkpoint inhibitors (ICIs) for these patients was still controversial. Methods: This retrospective study enrolled lung cancer patients harboring either EGFR or ERBB2 Ex20ins mutations. All the patients were treated with platinum-based chemotherapy plus ICIs, or platinum-based chemotherapy. The demographic features and clinical outcome of each patient were reviewed and analyzed. Results: When treated with immunochemotherapy, patients with EGFR/ERBB2 Ex20ins mutations (*n* = 31) had poor PFS compared with those without EGFR mutations (*n* = 141, 5.0 mon and 11.2 mon, *p* < 0.001). When compared with those with EGFR classic mutations who received immunotherapy as the salvage therapy (*n* = 24), these patients with EGFR/ERBB2 Ex20ins mutations had similar PFS (5.0 mon and 4.1 mon, *p* = 0.625), ORR (37.5% vs. 48.4%), and DCR (70.8% vs. 77.4%). In the patients with EGFR/ERBB2 Ex20ins mutations, the PFS of those treated with chemotherapy (*n* = 54) and those treated with immunochemotherapy (*n* = 31) was 6.5 mon vs. 5.0 mon (*p* = 0.066). In the EGFR Ex20ins subgroup, the PFS of addition of bevacizumab to chemotherapy (*n* = 20) and chemotherapy alone (*n* = 16) was 8.8 mon and 5.2 mon, respectively (*p* = 0.082) or immunochemotherapy (*n* = 15, 8.8 mon and 5.0 mon, *p* = 0.097). Similarly, in the ERBB2 subgroup, the combination of bevacizumab and chemotherapy achieved a numerically longer PFS over chemotherapy alone (9.1 mon and 4.5 mon, *p* = 0.253), but there was no statistical significance. Conclusions: This study showed that platinum-based chemotherapy plus ICIs had limited efficiency compared to platinum-based chemotherapy for patients with EGFR/ERBB2 Ex20ins. Chemotherapy plus bevacizumab may be a potential scheme for these patients.

## 1. Introduction

Lung cancer is the leading cause of cancer-related death in the world. Epidermal growth factor receptor (EGFR) mutations account for 15–25% of non-small cell lung cancer (NSCLC), and EGFR tyrosine kinase inhibitors (TKIs) such as gefitinib provide significant clinical benefit and improved progression-free survival (PFS) for these patients. Beyond classic mutations such as exon 19 deletion (19DEL) and exon21 mutation (L858R), exon 20 insertion (Ex20ins) comprises 4–10% of all EGFR mutations [1,2,3,4,5]. Besides, both EGFR (ERBB1) and ERBB2 belong to the ERBB family, with similar biological characteristics [6,7]. ERBB2 alterations comprise about 3% of all NSCLC patients, and ERBB2 Ex20ins is the most common mutation for ERBB2 alterations [8,9]. ERBB2 Ex20ins was the most common mutation for ERBB2 alterations [9,10]. However, the therapeutic effects of targeted therapy on either EGFR or ERBB2 Ex20ins are far from satisfactory. Historical data showed EGFR Ex20ins had an ORR of only 3–13% with EGFR-TKI in the first-line treatment [11,12].

Up to now, treatment-naïve patients with EGFR/ERBB2 Ex20ins are commonly treated with platinum-based chemotherapy [13,14,15]. Immune checkpoint inhibitors (ICIs) alone or combined with chemotherapy have become the standard of care for NSCLC without targeted mutations [16,17]. Several retrospective clinical studies have shown that a single agent of ICI had a low objective response rate (ORR) for EGFR/ERBB2 Ex20ins patients [11,18,19,20,21]. A few studies reported the effect of ICIs combined with chemotherapy (immunochemotherapy) on EGFR/ERBB2 Ex20ins, and more studies are urgently needed [22,23]. Our study aimed to explore the efficacy of immunochemotherapy for EGFR/ERBB2 Ex20ins.

## 2. Methods

### 2.1. Patients

**Figure 1:**
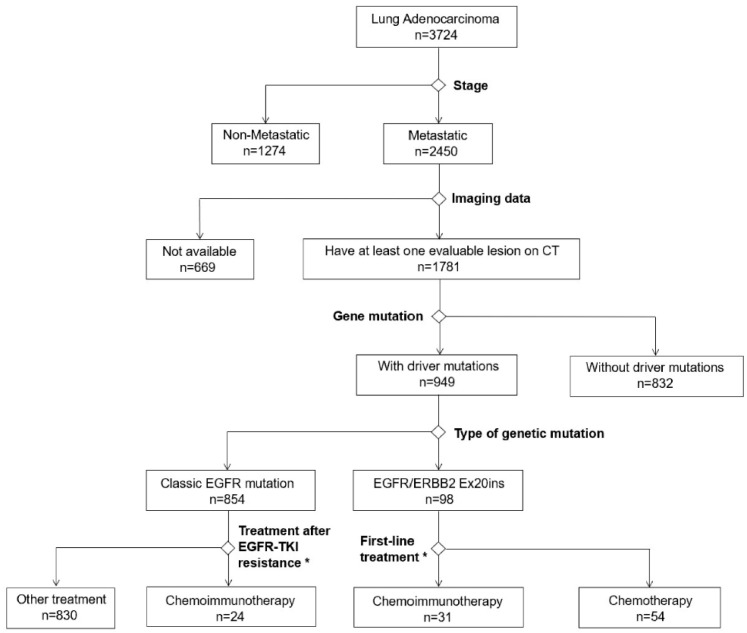
Flow chart of patient screening: * Receiving at least 1 cycle of chemotherapy alone or combined with ICIs.

This retrospective study was conducted at West China Hospital. Patients with pathologically confirmed NSCLC who had metastatic diseases between January 2018 and July 2021 were screened. Those receiving at least one cycle of platinum-based chemotherapy alone or combined with ICIs (immunochemotherapy) were enrolled. They must have at least one evaluable lesion on CT/MRI imaging. For the patients with no driver mutations or harboring EGFR/ERBB2 Ex20ins, only treatment-naïve ones were enrolled. For those with the classic EGFR mutation (19Del or L858R), chemo- or immunochemo-therapy was prescribed as salvage therapy after EGFR-TKI resistance (Figure 1). Patients with mixed small-cell lung cancer or those with other malignancies were excluded.

### 2.2. Treatments

For patients with squamous cancer, paclitaxel (Yangtze Inc., Beijing, China), nab-paclitaxel (Hengrui Inc., Lianyungang, China), or paclitaxel liposome (Luye Inc., Yantai, China) were prescribed. Gemcitabine (Lilly, Indianapolis, IN, USA) was also given to some patients. For those suffering from non-squamous cancer, pemetrexed (Hansoh Inc., Lianyungang, China) was the only regimen. Either cisplatin, carboplatin, or nedaplatin were used in the doublet regimen. ICIs included pembrolizumab (Merck, Rahway, NJ, USA), toripalimab (Junshi Inc., Shanghai, China), sintilimab (Innovent Inc., Beijing, China), and camrelizumab (Hengrui Inc., China). Immunochemotherapy, or chemotherapy, was prescribed according to the treating physicians’ discretion.

### 2.3. Genetic and PD-L1 Testing

EGFR/ERBB2 mutations were performed by polymerase chain reaction (PCR) with an authorized commercial kit (Amoyd Inc., Xiamen, China) before the year 2018. Later, in some patients, comprehensive genomic profiling was performed by NGS with 56 cancer-related gene panels covering the whole exons of the EGFR or ERBB2 gene at a mean coverage depth of >800×. PD-L1 expression was stained and assessed with the antibody 22C3 (Agilent Technologies, Santa Clara, CA, USA). The PD-L1 tumor proportion score (TPS) was calculated as the percentage of ≥100 viable tumor cells with complete or partial membrane staining. And TPS ≥ 1% was considered PD-L1 positive.

### 2.4. Response Assessment

The tumor response was evaluated every 2 months by the treating physician. The radiographic examinations included enhanced CT of the chest and upper abdomen, magnetic resonance imaging of the head, and bone scintigraphy. Tumor response was evaluated as complete response (CR), partial response (PR), stable disease (SD), or progression disease (PD), according to RECIST 1.1. PFS was defined as the duration from the start of the treatment to the date of disease progression, intolerable side effects, or death from any cause. ORR was defined as the proportion of patients achieving PR or CR. The disease control rate (DCR) was defined as the proportion of patients achieving SD, PR, or CR.

### 2.5. Statistical Analyses

Descriptive data were analyzed by the Chi-Square test or Fisher’s exact test. Cox proportional hazard regression models were applied to estimate the univariable, multivariable, and estimated hazard ratios (HRs). The Kaplan–Meier method was used to calculate the curves for the median PFS. Significant differences were determined by the log-rank test. All statistical tests were two-sided, and *p* < 0.05 was deemed to indicate statistical significance. Statistical analysis was performed using SPSS version 22.0 (IBM Inc., Chicago, IL, USA), and Kaplan–Meier curves were output by GraphPad Prism 7.00 (GraphPad Software Inc., San Diego, CA, USA).

## 3. Results

### 3.1. Genetic Aberrations in EGFR or ERBB2 Ex20ins

Totally, 51 patients harboring EGFR Ex20ins were enrolled. Among them, 15 were prescribed platinum-based chemotherapy plus ICI (group IC), and the rest 36 received platinum-based chemotherapy (group C). Some had available NGS data. The identified subtypes of EGFR Ex20ins mutations included A767_V769dup (*n* = 10), P772_H773dupPH (*n* = 4), S768_D770dup (*n* = 3), A763_Y764insFQEA (*n* = 1), N771_P772insThr (*n* = 1), and S768 _ 769delinsIL (*n* = 1). For ERBB2 Ex20ins, 16 (group IC) and 18 (group C) patients were collected. The most frequent genetic aberrations were Y772_A775dup (*n* = 4), G776delinsVC (*n* = 2), G778_P780dup (*n* = 2), A775_G776ins (*n* = 2), E770delinsEAYVM (*n* = 1), and S779_P780ins (*n* = 1).

### 3.2. EGFR/ERBB2 Ex20ins Showed Poor Responses to Immunochemotherapy

**Figure 2:**
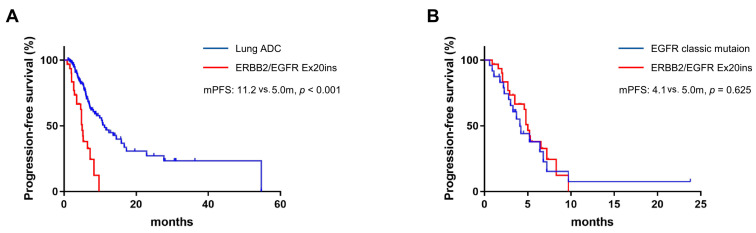
Patients with EGFR/ERBB2 Ex20ins had unfavorable PFS compared with those without driver mutations (**A**) and similar PFS compared with those harboring EGFR classic mutations (**B**) when receiving immunochemotherapy.

Totally, 31 patients harboring EGFR/ERBB2 Ex20ins (EGFR Ex20ins, *n* = 15; ERBB2 Ex20ins, *n* = 16) received immunochemotherapy. The efficacy of these patients was compared with that of another cohort of patients without targeted mutations (EGFR or ALK aberrations, *n* = 141). The latter cohort had more male patients and smokers (Table 1). Although the ORR did not differ between the cohorts (48.4% and 53.2%, *p* = 0.628), the DCR was significantly higher in the latter (77.4% vs. 91.5%, *p* = 0.024). PFS was poor in the Ex20ins group (median PFS 5.0 mon, 95%CI: 4.4–5.6 mon, and 11.2 mon, 95%CI: 8.9–13.7 mon, *p* < 0.001, Figure 2A).

Patients harboring EGFR classic mutations had poor outcomes after immunotherapy. Here, a total of 24 patients with EGFR classic mutations were enrolled (19DEL, *n* = 15, and L858R, *n* = 9), who were prescribed immunochemotherapy after failure of targeted therapy (Table 2). These patients had worse PS than those in the Ex20ins cohort (*p* < 0.001). In this cohort, the ORR and DCR were 37.5% and 70.8%, respectively (compared to 48.4% and 77.4% in the Ex20ins cohort). The PFS of these patients was similar to those with ERBB2/EGFR Ex20ins (4.1 mon and 5.0 mon, *p* = 0.625, Figure 2B).

### 3.3. Survival Analysis for EGFR/ERBB2 Ex20ins

**Figure 3:**
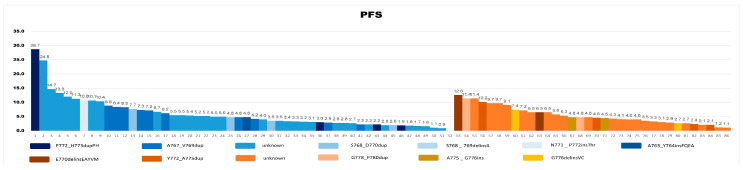
The PFS and the identified subtypes of gene mutations of each patient with EGFR Ex20ins (blue) or ERBB2 Ex20ins (orange) mutations who received either chemotherapy or immunochemotherapy.

**Figure 4:**
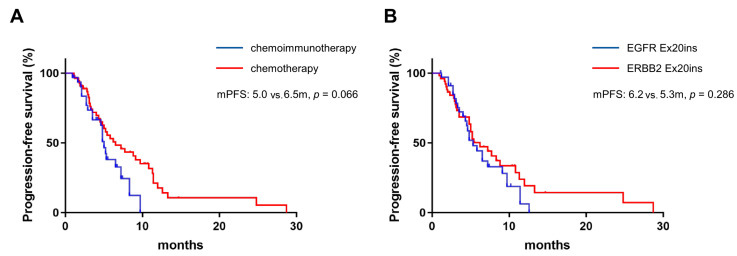
The PFS in patients with EGFR/ERBB2 Ex20ins treated by chemotherapy or immunochemotherapy (**A**). The PFS between EGFR Ex20ins and ERBB2 Ex20ins was comparable (**B**).

A total of 85 patients with EGFR/ERBB2 Ex20ins were analyzed (*n* = 54 in group C and *n* = 31 in group IC). The median follow-up was 10.2 mon (range 2.0–34.7 mon), with 69.4% experiencing disease progression at the time of analysis, and the median PFS was 5.4 mon (95% CI: 4.0–6.8 mon). The PFS and the identified subtypes of gene mutations in each EGFR/ERBB2 Ex20ins patient receiving chemotherapy or immunochemotherapy are shown in Figure 3. Patients in group C had a longer PFS (6.5 mon, 95% CI: 3.7–9.3 mon) than those in group IC (5.0 mon, 95% CI: 4.4–5.6 mon, *p* = 0.066, Figure 4A). However, this result was not statistically significant. EGFR Ex20ins and ERBB2 Ex20ins were not associated with a difference in PFS (6.2 mon and 5.3 mon, *p* = 0.286, Figure 4B). In univariate analysis, liver metastasis and poor PS were inversely associated with PFS (Table 3). In the multivariate analysis, only liver metastasis (HR, 2.053; 95% CI, 1.067–3.953) and immunochemotherapy (HR, 1.866; 95% CI, 1.037–3.354) were found to be significantly associated with PFS.

### 3.4. Chemo- or Immunochemo-Therapy for EGFR Ex20ins

**Figure 5:**
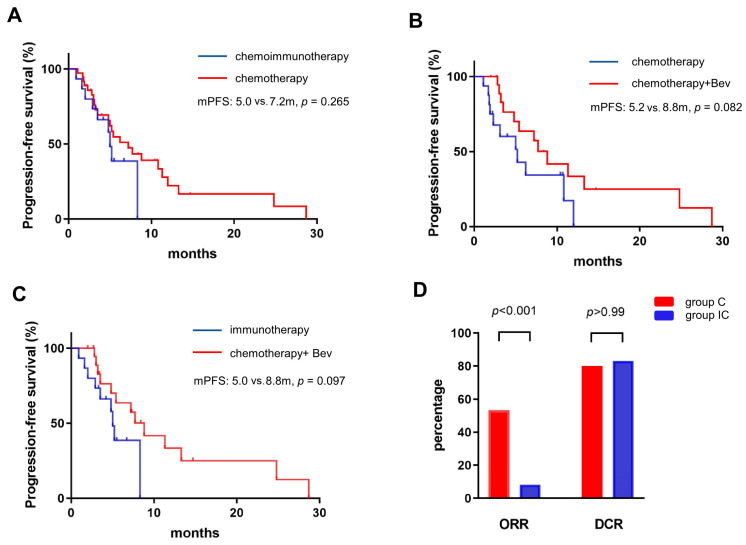
In EGFR Ex20ins patients, either chemo- or immunochemo-therapy achieved a similar PFS (**A**). The addition of bevacizumab to chemotherapy achieved a longer PFS over chemotherapy alone (**B**) or immunochemotherapy (**C**). ORR and DCR of EGFR Ex20ins patients in the chemotherapy group and immunochemotherapy (**D**).

For EGFR Ex20ins, the efficacy of immunochemotherapy (group IC) and chemotherapy (group C) was compared. Immunochemotherapy achieved a higher ORR (53.3%, 8/15, and 8.3%, 3/36, *p* < 0.001) but a similar DCR (80.0% and 83.3%, *p* > 0.99, Figure 5D). Immunochemotherapy did not show superior PFS over chemotherapy (5.0 mon and 7.2 mon, *p* = 0.265; Figure 5A). In group IC, 8 patients remained PR and 4 remained SD. In group C, 3 and 27 patients remained PR and SD.

In group C, patients received chemotherapy with (*n* = 20) or without (*n* = 16) bevacizumab. The addition of bevacizumab raised ORR from 6.3% to 10.0% and DCR from 81.3% to 90.0%, respectively. Compared with immunochemotherapy, the combination of bevacizumab and chemotherapy achieved a lower ORR (10.0% vs. 53.3%, *p* = 0.008). Importantly, although the results were not statistically significant, longer PFS was observed when bevacizumab was used over chemotherapy alone (8.8 mon and 5.2 mon, *p* = 0.082, Figure 5B) or immunochemotherapy (8.8 mon and 5.0 mon, *p* = 0.097, Figure 5C).

### 3.5. Chemo- or Immunochemo-Therapy for ERBB2 Ex20ins

**Figure 6:**
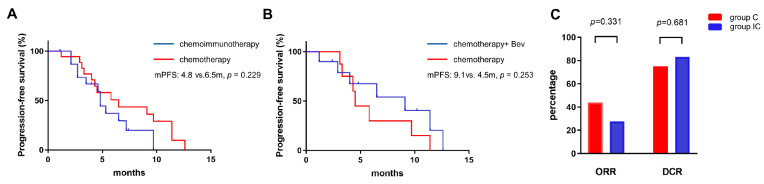
In ERBB2 Ex20ins patients, either chemo- or immunochemo-therapy achieved a similar PFS (**A**). The combination of bevacizumab and chemotherapy did not show a longer PFS over chemotherapy alone (**B**). ORR and DCR of ERBB2 Ex20ins patients in the chemotherapy group and immunochemotherapy (**C**).

For ERBB2 Ex20ins, the ORR of group C and group IC was 43.8% (7/16) and 27.8% (5/18), respectively (*p* = 0.331). And the DCR was 75% (12/16) and 83.3% (15/18), respectively (*p* = 0.681, Figure 6C). Immunochemotherapy led to even numerically worse PFS (4.8 mon, 95% CI: 4.4–5.2 months) compared to chemotherapy alone (6.5 mon, 95% CI: 3.1–9.9 mon, *p*= 0.229, Figure 6A). In group C, 7 patients received bevacizumab, and the PFS was 9.1 mon (95% CI: 2.6–15.6 mon), while the other 11 patients had a PFS of only 4.5 mon (95% CI: 4.0–5.0 mon, *p* = 0.253, Figure 6B).

## 4. Discussion

In this study, the efficacy of ICIs on patients harboring EGFR/ERBB2 Ex20ins was explored. ICIs, even in combination with chemotherapy, showed poor efficacy in these patients. They had worse DCR and shorter PFS when compared with those suffering from adenocarcinoma without driver mutations. And we found the efficacy of ICI in these patients was similar to that of those harboring the classic EGFR mutation (19Del or L858R). In these patients, the addition of ICI to chemotherapy led to a higher ORR but an unfavorable PFS. Alternatively, chemotherapy plus bevacizumab may be a better scheme.

The optimal treatments for EGFR/ERBB2 Ex20ins remained largely unknown. Previously, platinum-based doublet chemotherapy was widely used. However, the outcome of chemotherapy (median PFS of 4.2 mon–7.6 mon) was quite dissatisfying [13,14,24]. ICI treatment was proposed recently. ICI monotherapy was tested in several retrospective clinical studies. Guisier et al. first reported immunotherapy for NSCLC with an ERBB2 mutation in 2018, and 23 patients received ICIs in later-line treatment. Six of them achieved PR and five achieved SD, while the median PFS was only 2.2 months [25]. Metro et al. enrolled 30 advanced NSCLC patients with EGFR Ex20ins; 15 patients received chemotherapy, and the other 15 received immunotherapy or chemo-immunotherapy. The ORR of the immunotherapy group was only 6.7% (1/15) [17]. Recently, another study enrolled 48 patients with EGFR (*n* = 6) and ERBB2 (*n* = 14) Ex20ins mutations. The median PFS of EGFR and ERBB2 mutations was 4.8 months and 3.6 months, respectively [6]. Saalfeld et al. evaluated 32 patients with ERBB2 mutations (including Ex20ins). All received ICI single agents in the later-line treatment, and the ORR was 16% [21]. From these studies, ICI monotherapy had poor efficacy for EGFR/ERBB2 Ex20ins.

ICIs plus chemotherapy bring survival benefits to advanced lung adenocarcinoma without driver mutations [16,23,26,27]. However, the efficacy of this combination for EGFR/ERBB2 Ex20ins was still unknown. In one study, 3 patients with ERBB2 Ex20ins received this combination, with 1 PR, 1 SD, and 1 PD [22]. In another study, in 13 patients with ERBB2 Ex20ins, the combination achieved an ORR of 31% and a median PFS of 8.0 months [10]. For EGFR Ex20ins, 12 patients received this combination, and the median PFS was 7.0 months [28]. These anecdotal, small-scale studies implied that the combination of immunochemotherapy might improve the prognosis of ERBB2/EGFR Ex20ins. It is worth nothing that no control group was included in these studies. In Table 4 the studies on ERBB2/EGFR Ex20ins were summarized. Generally, ICI monotherapy had a shorter PFS, and either chemo- or immunochemotherapy had a longer PFS. In this study, patients with ERBB2/EGFR Ex20ins receiving immunochemotherapy achieved a PFS of 5.0 mon, in good accordance with other reports. However, a numerically longer PFS was observed by chemotherapy alone (6.5 mon). Besides, the PFS of ERBB2/EGFR Ex20ins was similar to that of EGFR classic mutations (5.0 mon and 4.1 mon, *p* = 0.625), which was notorious for its unresponsiveness to immunotherapy. Our study argued against the combination of immunochemotherapy for EGFR/ERBB2 Ex20ins.

The tumor microenvironment determined the efficacy of immunotherapy. In one study, a high proportion (75.9%) of patients with EGFR Ex20ins (*n* = 141) had negative expression of PD-L1. Among these patients, the median TMB was only 4.6/Mb (*n* = 36) [30]. Similarly, a low positive expression of PD-L1 (19.0%) was reported in patients with ERBB2 Ex20ins (*n* = 21). Again, the average TMB was only 3.3/Mb [19]. In another interesting study, similar TMB was found in EGFR Ex20ins (*n* = 260) and EGFR classic mutations (*n* = 1318, 3.6/Mb and 3.6/Mb, *p* = 0.31) [2]. The negative expression of PD-L1 and low TMB in EGFR/ERBB2 Ex20ins might explain the poor outcome of ICI.

In the subgroup analysis of EGFR Ex20ins, the addition of bevacizumab to chemotherapy outperformed chemo- or immunochemo-therapy in terms of PFS. In the subgroup of ERBB2 Ex20ins, similarly, adding bevacizumab led to a numerically longer PFS. The statistical insignificance was most likely due to the small sample size. This was interesting because this observation implied that bevacizumab might be a potential option for the patients with EGFR/ERBB2 Ex20ins. Larger studies were warranted to confirm this observation.

In recent years, there have been many new developments in the research and development of drugs targeting EGFR Ex20ins in the field of NSCLC. Mobocertinib is a small-molecule TKI specifically designed for EGFR Ex20ins and ERBB2 Ex20ins [29,31]. However, the Phase III EXCLAIM-2 confirmatory study of mobocertinib did not meet the primary study endpoint [32]. Amivantamab, approved by the FDA, had an ORR of 40% and a median PFS of 8.3 months in CHRYSALIS studies [33]. The confirmatory study for amivantamab, the PAPILLION study, randomized patients with EGFR Eex20ins to the amivantamab plus chemotherapy group or the chemotherapy alone group. The median PFS for patients in the amivantamab plus chemotherapy group and chemotherapy alone group was 11.4 and 6.7 months, respectively. Adding amivantamab to chemotherapy was associated with a relatively significant 60% reduction in risk for disease progression or death (HR = 0.40) and an even greater difference in 18-month PFS of 31% vs. 3% [34]. This treatment strategy is expected to become the clinical standard of care. In addition to these two drugs, there are many other drugs targeting EGFR Ex20ins in clinical trials, such as CLN081/TAS6417 (zipalertinib) and DZD9008 (sunvozertinib).

There are several limitations to this study. Firstly, it was based on a retrospective cohort from a single institute. Secondly, the sample size was not enough, and the statistical results may have been biased. Finally, in this study, only the efficacy of immunochemotherapy in this subset of patients was taken as this study endpoint, and the effect of this treatment strategy on survival time was not deeply explored.

To our knowledge, this study was the first to compare chemo- and immunochemo-therapy for advanced NSCLC with EGFR/ERBB2 Ex20ins mutations. Overall, our results showed that in these patients, the addition of ICI failed to improve PFS, and bevacizumab might be a better treatment option.

## Figures and Tables

**Table 1:** Clinical characteristics of EGFR/ERBB2, EGFR classic mutations, and lung adenocarcinoma with platin-based chemotherapy plus ICIs.

Characteristics	ERBB2/EGFR Ex20ins	EGFR Classic Mutations	Adenocarcinoma *	
	*n* = 31	*n* = 24	*n* = 141	*p*
Age				
≥60	13 (41.9%)	11 (45.8%)	88 (62.4%)	0.056
<60	18 (58.1%)	13 (54.2%)	53 (37.6%)	
Gender				
Male	17 (54.8%)	13 (54.2%)	112 (79.4%)	0.002
Female	14 (45.2%)	11 (45.8%)	29 (20.6%)	
ECOG				
0	22 (71.0%)	5 (20.8%)	100 (70.9%)	0.001
≥1	9 (29.0%)	19 (79.2%)	41 (19.1%)	
Smoking				
Yes	4 (12.9%)	4 (16.7%)	71 (50.4%)	0.001
No	27 (87.1%)	20 (83.3%)	70 (49.6%)	
PD-L1 expression				
≥1%	12 (38.7%)	14 (58.3%)	70 (49.6%)	0.553
Negative	7 (22.6%)	3 (12.5%)	19 (13.5%)	
Unknown	12 (38.7%)	7 (29.2%)	52 (36.9%)	
Previous treatment				
TKI	4 (12.9%)	24 (100%)	0	
No	27 (87.1%)	0	141 (100%)	
Previous treatment				NA
Chemo/ICI	0	0	0	
No	31 (100%)	24 (100%)	141 (100%)	
ICIs				NA
PD-1	31 (100%)	24 (100%)	141 (100%)	
PD-L1/CTLA-4	0	0	0	
Chemotherapy				NA
Platin-based	31 (100%)	24 (100%)	141 (100%)	
other	0	0	0	
Brain metastases				0.085
Yes	8 (25.8%)	11 (45.8%)	34 (24.1%)	
No	23 (74.2%)	13 (54.2%)	107 (75.9%)	

* Lung adenocarcinoma without ERBB2/EGFR Ex20ins and EGFR classic mutation.

**Table 2:** Clinical characteristics of patients with EGFR/ERBB2 Ex20ins.

Characteristics	Patients	EGFR EX20ins IC	EGFR EX20ins C	*p*	ERBB2 EX20ins IC	ERBB2 EX20ins C	*p*
	85	15 (17.6%)	36 (42.4%)		16 (18.8%)	18 (21.2%)	
Age				0.971			0.703
≥60	36	8 (53.3%)	19 (52.8%)		5 (31.2%)	4 (22.2%)	
<60	49	7 (46.7%)	17 (47.2%)		11 (68.8%)	14 (77.8%)	
Gender				0.078			>0.99
Male	39	9 (60.0%)	12 (33.3%)		8 (50.0%)	10 (55.6%)	
Female	46	6 (40.0%)	24 (66.7%)		8 (50.0%)	8 (44.4%)	
ECOG				0.513			0.681
0	57	12 (80.0%)	25 (69.4%)		10 (62.5%)	10 (55.6%)	
≥1	28	3 (20.0%)	11 (30.6%)		6 (37.5%)	8 (44.4%)	
Smoking history				>0.99			0.125
Yes	17	2 (13.3%)	6 (16.7%)		2 (12.5%)	7 (38.9%)	
No	68	13 (86.7%)	30 (83.3%)		14 (87.5%)	11 (61.1%)	
Pathology				>0.99			>0.99
adenocarcinoma	83	15 (100%)	35 (97.2%)		15 (93.8%)	18 (100%)	
Other	2	0	1 (2.8%)		1 (6.2%)	0	
Treatment *							NA
Pembrolizumab + chemo	9	3 (20.0%)			6 (37.5%)		
Toripalimab + chemo	1	0			1 (6.2%)		
Sintilimab + chemo	5	2 (13.3%)			3 (18.8%)		
Camrelizumab + chemo	13	8 (53.3%)			5 (31.2%)		
Tislelizumab + chemo	3	2 (13.3%)			1 (6.2%)		
AC/P+ Bev	21		13 (36.1%)			8 (44.4%)	
AC/P	28		20 (55.6%)			8 (44.4%)	
TC/P+ Bev	2		2 (5.6%)			0	
TC/P	3		1 (2.8%)			2 (11.1%)	
Brain metastases				0.749			0.681
Yes	23	4 (26.7%)	12 (33.3%)		4 (25.0%)	3 (16.7%)	
No	62	11 (73.3%)	24 (66.7%)		12 (75.0%)	15 (83.3%)	
Liver metastases				0.657			>0.99
Yes	16	1 (6.7%)	5 (13.9%)		5 (31.2%)	5 (27.8%)	
No	69	14 (93.3%)	31 (86.1%)		11 (68.8%)	13 (72.2%)	

Abbreviations: Line of Treatment *: First-line chemotherapy or chemoimmunotherapy. IC, immunotherapy + chemotherapy; C, chemotherapy; AC/P, pemetrexed + carboplatin/cisplatin; Bev, Bevacizumab; TC/P, paclitaxel + carboplatin/cisplatin.

**Table 3:** Univariate and multivariate analyses of ERBB2/EGFR Ex20ins.

	Univariate Analysis		Multivariate Analysis	
Factors	*p* Value	HR (95%CI)	*p* Value	HR (95%CI)
Age (<60 y vs. ≥60 y)	0.289	1.402 (0.840–2.340)		
Gender (male vs. female)	0.466	0.910 (0.546–1.517)		
Smoking history (yes vs. no)	0.941	0.912 (0.492–1.691)		
PS (0 vs. ≥1)	0.044	0.673 (0.386–1.173)	0.064	0.599 (0.348–1.030)
Ex20ins (EGFR vs. ERBB2)	0.286	0.763 (0.451–1.293)		
Brain metastasis (yes vs. no)	0.340	1.315 (0.721–2.400)		
Liver metastasis (yes vs. no)	0.029	1.965 (0.883–4.372)	0.031	2.053 (1.067–3.953)
PD-L1 expression (0% vs. ≥1%)	0.410	0.767 (0.394–1.496)		
Treatment (PCI vs. PC)	0.066	1.592 (0.888–2.853)	0.037	1.866 (1.037–3.354)

**Table 4:** Previous studies for ERBB2/EGFR Ex20ins. NR: not reported.

	Patients	Mutation	Treatment	Line	mPFS	mOS	ORR	DCR
Shah et al. [14]	*N* = 18	EGFR Ex20ins	Platinum-based chemotherapy	First/second	7.1 m	3.2 y	39%	NR
Xu et al. [15]	*N* = 77	EGFR Ex20ins	Pemetrexed-based chemotherapy	First	5.5 m	25 m	41.56%	75.32%
Chelabi et al. [16]	*N* = 27	EGFR Ex20ins	Chemotherapy	First	6.5 m	NR	41%	82%
Xu et al. [25]	*N* = 37	ERBB2 Ex20ins	Chemotherapy	First	5.5 m	NR	NR	NR
Wang et al. [29]	*N* = 49	EGFR Ex20ins	Platinum-based chemotherapy	First	7.6 m	19.9 m	NR	NR
Lau et al. [7]	*N* = 6	EGFR Ex20ins	PD-1/PD-L1	First/second/third	4.8 m	NR	50%	66.7%
Tian et al. [11]	*N* = 13	ERBB2 Ex20ins	Chemo-ICI	First/second	8.0 m	NR	31%	77%
Metro et al. [19]	*N* = 15	EGFR Ex20ins	ICI or chemo-ICI	First/second/third	2.0 m	5.3 m	6.7%	13.3%
Chen et al. [20]	*N* = 9	EGFR Ex20ins	PD-1/PD-L1	NR	NR	NR	22.2%	NR
Chen et al. [20]	*N* = 6	ERBB2 Ex20ins	PD-1/PD-L1	NR	NR	NR	0%	NR
Hastings et al. [21]	*N* = 28	EGFR Ex20ins	ICI	First/second/third	1.9 m	5.5 m	15.2%	32.1%
Choudhury et al. [30]	*N* = 12	EGFR Ex20ins	Chemo-ICI	First/second/third	7 m	NR	NR	NR

## Data Availability

The full original source data can be accessed atin https://www.jianguoyun.com/p/DQ7Uz_0Q7oeDChjS78IEIAA, accessed on 1 December 2022.
